# EGFR Inhibition by Cetuximab Modulates Hypoxia and IFN Response Genes in Head and Neck Squamous Cell Carcinoma

**DOI:** 10.1158/2767-9764.CRC-22-0443

**Published:** 2023-05-22

**Authors:** Ritu Chaudhary, Robbert J.C. Slebos, Leenil C. Noel, Feifei Song, Maria I. Poole, Dirk S. Hoening, Juan C. Hernandez-Prera, Jose R. Conejo-Garcia, Jose A. Guevara-Patino, Xuefeng Wang, Mengyu Xie, Aik Choon Tan, Christine H. Chung

**Affiliations:** 1 Department of Head and Neck-Endocrine Oncology, Moffitt Cancer Center, Tampa, Florida.; 2 Department of Pathology, Moffitt Cancer Center, Tampa, Florida.; 3 Department of Immunology, Moffitt Cancer Center, Tampa, Florida.; 4 Department of Biostatistics and Bioinformatics, Moffitt Cancer Center, Tampa, Florida.; 5 Department of Oncological Sciences, Huntsman Cancer Institute, University of Utah, Salt Lake City, Utah.

## Abstract

Head and neck squamous cell carcinoma (HNSCC) has one of the most hypoxic and immunosuppressive tumor microenvironments (TME) among solid tumors. However, there is no proven therapeutic strategy to remodel the TME to be less hypoxic and proinflammatory. In this study, we classified tumors according to a Hypoxia-Immune signature, characterized the immune cells in each subgroup, and analyzed the signaling pathways to identify a potential therapeutic target that can remodel the TME. We confirmed that hypoxic tumors had significantly higher numbers of immunosuppressive cells, as evidenced by a lower ratio of CD8^+^ T cells to FOXP3^+^ regulatory T cells, compared with nonhypoxic tumors. Patients with hypoxic tumors had worse outcomes after treatment with pembrolizumab or nivolumab, anti-programmed cell death-1 inhibitors. Our expression analysis also indicated that hypoxic tumors predominantly increased the expression of the EGFR and TGFβ pathway genes. Cetuximab, an anti-EGFR inhibitor, decreased the expression of hypoxia signature genes, suggesting that it may alleviate the effects of hypoxia and remodel the TME to become more proinflammatory. Our study provides a rationale for treatment strategies combining EGFR-targeted agents and immunotherapy in the management of hypoxic HNSCC.

**Significance:**

While the hypoxic and immunosuppressive TME of HNSCC has been well described, comprehensive evaluation of the immune cell components and signaling pathways contributing to immunotherapy resistance has been poorly characterized. We further identified additional molecular determinants and potential therapeutic targets of the hypoxic TME to fully leverage currently available targeted therapies that can be administered with immunotherapy.

## Introduction

Head and neck squamous cell carcinoma (HNSCC) is a heterogeneous disease that includes tumors originating in the oral cavity, oropharynx, hypopharynx, or larynx. HNSCC is the sixth most common cancer worldwide (1). Common risk factors include tobacco use, excessive alcohol consumption, and human papillomavirus (HPV) infection (2, 3). The treatment options for HNSCC include surgery, radiation, chemotherapy, immunotherapy, targeted therapy, and various combinations. Even with multimodality therapies, the 5-year mortality rate is approximately 40%–50%, although HPV-associated HNSCC has better survival than smoking-related HNSCC (4, 5). A better understanding of the molecular mechanisms underlying these poor outcomes may help in the development of novel treatments.

The tumor microenvironment (TME) plays an important role in tumor survival, proliferation, epigenetics, and immune evasion. HNSCC has a highly complex TME comprising heterogeneous cell types with multidirectional cross-talk among the tumor, immune, and stromal components. A hypoxic TME is accompanied by increased genomic instability (6, 7). Hypoxia induces immunosuppression by directly inhibiting T-cell proliferation and effector cytokine production, as well as indirectly by creating a hostile TME for T cells through metabolic competition, upregulation of coinhibitory receptors, and/or recruitment of immunosuppressive cells, such as myeloid-derived suppressor cells (MDSC; refs. 8–10). Exposure to tobacco smoke aggravates tissue hypoxia in human HNSCCs (11). Similarly, cigarette smoke extract increases HIF-1 activity in human cells, which in turn induces the expression of hypoxia-related genes (12).

When 8,006 tumors were characterized for the presence of hypoxia based on their gene expression, HNSCC was one of the most hypoxic tumors among the 19 distinct tumor types analyzed (13). A recent study by Brooks and colleagues classified HNSCCs using a 54-gene Hypoxia-Immune signature (14) and identified three subgroups:(i) hypoxia^high^/immune^low^ (Hypoxia subgroup), mainly comprised of HPV-negative cases lacking immune-related markers; (ii) hypoxia^low^/immune^high^ (Immune subgroup), enriched for HPV-positive cases, comprising a high percentage of immune-related genes; and (iii) mixed signatures (Mixture subgroup), consisting of both HPV-positive and HPV-negative cases (14). Their data suggest that tobacco exposure is associated with a hypoxic TME, which plays a crucial role in determining tumor progression, immune surveillance, treatment responses, and survival (14, 15).

Patients with HNSCC who actively smoke during treatment have worse survival rates than never smokers and former smokers (15, 16). In our previous work, we demonstrated that the TME of current smokers has lower numbers of cytotoxic CD8^+^ T cells compared with neversmokers and former smokers, suggesting an immunosuppressive TME in tumors from current smokers (15). Gene expression analyses of these tumors showed that immunosuppression was partly due to the suppression of the IFNα and IFNγ response pathways in current smokers. Regulation of the IFN response pathway is complex, as IFNs can mediate both antitumor and protumor effects (17, 18). Apart from smoking, there are several TME stress factors, including hypoxia and tumor-expressed inflammatory cytokines such as IL1, that can contribute to the dysregulation of IFN pathways (15, 19). Hypoxia induces downregulation of the type I IFN pathway owing to suppressed gene transcription and decreased chromatin accessibility (20). Hypoxia inhibits the upregulation of IFNγ-induced MHC class I expression and that of the IFNγ-stimulated chemokines, *CXCL9* and *CXCL10* (21).

Hypoxia-associated immunosuppression in HNSCC is clinically significant because the current standard of care for recurrent and/or metastatic HNSCC is the inhibition of programmed cell death-1 (PD-1) using antibodies such as pembrolizumab and nivolumab (22, 23). Although these immune-targeted agents are highly effective in selected patients, particularly in patients with high expression of programmed cell death ligand-1 (PD-L1) quantified by combined positive score (CPS), most patients eventually develop treatment resistance (22). In this study, we identified HNSCCs with a hypoxic TME, based on a previously developed Hypoxia-Immune signature (14). We further characterized the molecular determinants and potential therapeutic targets of hypoxic TME, as well as proinflammatory TME, to fully leverage the currently available immune-targeted therapies.

## Materials and Methods

### Moffitt HNSCC Dataset

Patients with HNSCC at the H. Lee Moffitt Cancer Center & Research Institute were identified through three Institutional Review Board (IRB)-approved studies: Total Cancer Care (MCC#14690; Table 1), Epidemiology of Head and Neck Cancer Study (MCC#17041; Table 1), and Evaluation of The Tumor and Its Microenvironment in Head and Neck Cancer Patients (MCC#18754; Table 1 and Table 2, retrospective study, written consent waived). IRB approval was obtained in accordance with the Department of Health and Human Services Federal Policy for the Protection of Human Subjects (U.S. Common Rule). Tumor tissues from 224 HPV-negative patients were identified and sequenced at the Hudson Alpha Institute for Biotechnology as part of the Moffitt Cancer Center Oncology Research Information Exchange Network (ORIEN) AVATAR network. RNA sequencing (RNA-seq) library preparation and data analysis were described previously (15).

**TABLE 1:** Patient characteristics in Moffitt cohort

	Hypoxia/Immune status				
	Hypoxia	Mixture	Immune	Total	*P* ^a^
Median age (range)	59 (30–84)	63 (23–87)	64 (44–83)	62 (21–87)	
Gender Male Female	57 19	73 27	33 19	163 65	0.334
Disease site Oral cavity Larynx Oropharynx Hypopharynx Nasal sinus Unknown primary	46 20 5 4 0 1	64 27 5 3 0 1	35 9 6 1 1 0	145 56 16 8 1 2	0.593
Clinical stage I II III IV Unknown stage	16 10 8 35 7	22 10 12 40 16	16 8 6 16 6	54 28 26 91 29	0.524
Smoking history Never smoker Former smoker Current smoker	17 28 31	13 35 52	15 21 16	45 84 99	0.064
Total	76	100	52	228	

^a^Fisher exact test.

**TABLE 2:** Disease control given pembrolizumab or nivolumab monotherapy in Moffitt retrospective study cohort

	Hypoxia	Mixture	Immune
No disease control	14	15	7
Disease control	2	3	7

NOTE: No disease control included patients with progressive disease and/or stable disease. Disease control included patients with complete response and/or partial response. *P* = 0.021, Cochran–Armitage test for trend.

### Public HNSCC Datasets

Normalized RNA-seq data for The Cancer Genome Atlas (TCGA) HNSCC cohort were publicly available and downloaded from cBioportal (https://cbioportal-datahub.s3.amazonaws.com/HNSCC_tcga_pan_can_atlas_2018.tar.gz). Of the 523 samples, there were no clinical data available for four samples, and one additional sample was missing survival data (Supplementary Table S1). Aligned RNA-seq data (BAM files) for the Clinical Proteomic Tumor Analysis Consortium (CPTAC) cohort were downloaded from GDC and quantified using the RSEM pipeline, as described previously (24). In the CPTAC cohort, among the 109 tumor samples in the CPTAC cohort with sequencing data, one sample had missing clinical information (Supplementary Table S2). All RNA-seq data were transformed using log_2_(TPM+1). Window-of-opportunity trial data by Schmitz and colleagues were downloaded from the Gene Expression Omnibus (NCBI GEO) with accession number GSE109756 (14). These samples were profiled using the Affymetrix Human Genome U133 Plus 2.0. Raw microarray data samples were normalized using the Robust Multi-array Average method using Affymetrix Power Tools, as published previously (25). Multiple probe sets representing the same genes were collapsed using a probe with the maximum gene expression. In this study, patients were treated with three doses of cetuximab 15 days prior to surgery.

### Heatmaps

Heatmaps were created with the Morpheus tool (http://software.broadinstitute.org/morpheus) using log_2_-normalized RNA-seq counts of common genes across the HNSCC datasets. Supervised hierarchical clustering was performed on the z-transformed data using one-minus Spearman rank correlation and complete linkage scores. Hypoxia, Immune, and Mixture subgroups were determined by manual examination of the resulting dendrograms.

### Multiplex IHC

Tissue microarray (TMA) blocks were prepared from three independent punches of tumor specimens from each patient. A total of 121 tumor cores (including 81 primary, 28 recurrent primary, and 12 lymph node metastases) were embedded in each of the three TMA blocks. Of the 121 total cases, sequencing data were available for 90 samples (including 63 primary, 20 recurrent primary, and seven lymph node metastases). For multiplex IHC (mIHC), formalin-fixed paraffin-embedded (FFPE) TMA blocks were sectioned at 4 μm onto charged AutoFrost IHC-enhanced coated hydrophilic slides. The slides were deparaffinized and washed with alcohol, followed by hematoxylin staining. The detailed protocol has been described previously (26). Two slides were stained for each TMA replicate. The first slide was stained sequentially with the following lymphoid markers: hematoxylin #SH26 (Thermo Fisher Scientific), CD4 #M731001 (DAKO), CD56 #M730401 (DAKO), GrzB #M723501 (DAKO), PD-1 #86163 (Cell Signaling Technology), FOXP3 #ab20034 (Abcam), PD-L1 #13684 (Cell Signaling Technology), CD8 #M710301 (DAKO), CD69 #ab23396 (Abcam), CD3 #M725401 (DAKO), CD20 #M075501 (DAKO), CD103 #ab227697 (Abcam), CD45 #13917 (Cell Signaling Technology), and PCK #M351501-2 (DAKO). The second slide was stained sequentially with the following myeloid markers: hematoxylin, CD14 #75181 (Cell Signaling Technology), Ki-67 #M724001 (DAKO), CD33 #ab199432 (Abcam), CD15 #M363101 (DAKO), DC-SIGN #ab59192 (Abcam), tryptase (TRP) #ab151757 (Abcam), CD66b #ab197678 (Abcam), CD68 #76437 (Cell Signaling Technology), CSF1R #ab183316 (Abcam), CD163 #ab182422 (Abcam), MHC-II #ab157210 (Abcam), HLA-DR #ab20181 (Abcam), CD206 #91992 (Cell Signaling Technology), and PCK #M351501-2 (DAKO). An independent TMA slide was stained with hematoxylin, hypoxia marker CAIX #CAIX-L-CE (Leica), and PCK #M351501-2 (DAKO). Differences in immune cell counts between the three subgroups were determined using CellProfiler (27), and the R-project code was used for statistical analyses, as described previously (26).

### PD-L1 CPS

A subset of archived FFPE tumors in the Moffitt cohort were obtained, and PD-L1 expression in each whole tumor slide was determined using PD-L1 #13684 (Cell Signaling Technology). The PD-L1 CPS was determined by counting the number of PD-L1–positive cells (tumor, lymphocytes, and macrophages) divided by the total number of viable cells multiplied by 100 as described previously (22). In addition, existing PD-L1 CPS data obtained as a standard of care were collected by a retrospective chart review of the Moffitt cohort. For the standard-of-care PD-L1 CPS, the PD-L1 staining was obtained using 22C3 pharmDx assay (Agilent Technologies) as described previously (22).

### Gene Set Enrichment Analysis

To identify different molecular subgroups, we performed gene set enrichment analysis (GSEA) against Hallmark of Cancer signatures using the GSEA toolkit version 4.1.0 (28). The FDR for the enriched pathways was estimated by performing 1,000 gene set permutations, and FDR < 0.05 was used as the threshold for determining pathway/gene set differences as significant. Two of the enriched Hallmark pathways from the Immune versus Hypoxia samples were hypoxia and TGFβ signaling. To study the possible relationships between these two pathways, we selected core-enriched genes from the GSEA and analyzed their interaction patterns using the STRING database (www.string-db.org) using all interaction sources except for text mining and co-occurrence.

### TIMEx

To compute gene expression scores, we used TIMEx, a novel TME deconvolution method for bulk transcriptomics using pan-cancer single-cell RNA-seq signatures (29), to deconvolute individual patient gene expression profiles. The underlying algorithm for deconvoluting the signatures is based on a single-sample GSEA, as described previously (29). The EGFR signaling pathway gene list was downloaded from Molecular Signatures Database (MSigDB) Gene Ontology: BP_EPIDERMAL_GROWTH_FACTOR_RECEPTOR_SIGNALING_PATHWAY (https://www.gsea-msigdb.org/gsea/msigdb/index.jsp). The E scores for patients within a specific molecular subtype were averaged to obtain a score for each cell type. The TIMEx EGFR pathway and hypoxia gene signature scores were calculated for all three HNSCC cohorts. Similarly, the TIMEx IFNγ score and hypoxia score were calculated for the Schmitz and colleagues dataset (14). The relationships between these scores were determined using the stat package in R (www.r-project.org).

### Statistical Analysis

Survival analyses were performed using the “survival” package in R to visualize survival curves using Kaplan–Meier plots and log-rank tests for significance. mIHC comparisons among the three subgroups were performed using ANOVA and Tukey *post hoc* tests, where appropriate. To determine trends from Immune to Hypoxia status, we used the Cochran–Armitage test to compare current smokers versus never smokers. Similarly, we used the Cochran–Armitage test for trend to compare patient responses to immunotherapy from Hypoxia to Immune status. A one-sided Wilcoxon signed-rank test was used to determine decreases in the Hypoxia signature and increases in the IFN gene signature between pre- and post-cetuximab samples. Associations between TIMEx EGFR and hypoxia scores were tested using Pearson correlation coefficient.

### Data Availability Statement

Normalized RNA-seq data for TCGA HNSCC cohort were publicly available and were downloaded from cBioportal (https://cbioportal-datahub.s3.amazonaws.com/HNSCC_tcga_pan_can_atlas_2018.tar.gz). Aligned RNA-seq data (BAM files) for the CPTAC cohort was downloaded from the GDC and quantified using the RSEM pipeline as described previously. The Moffitt Cohort raw data are available at dbGaP Study Accession: phs001994.v1.p1.

## Results

### Determination of HNSCCs with Hypoxia

**FIGURE 1:**
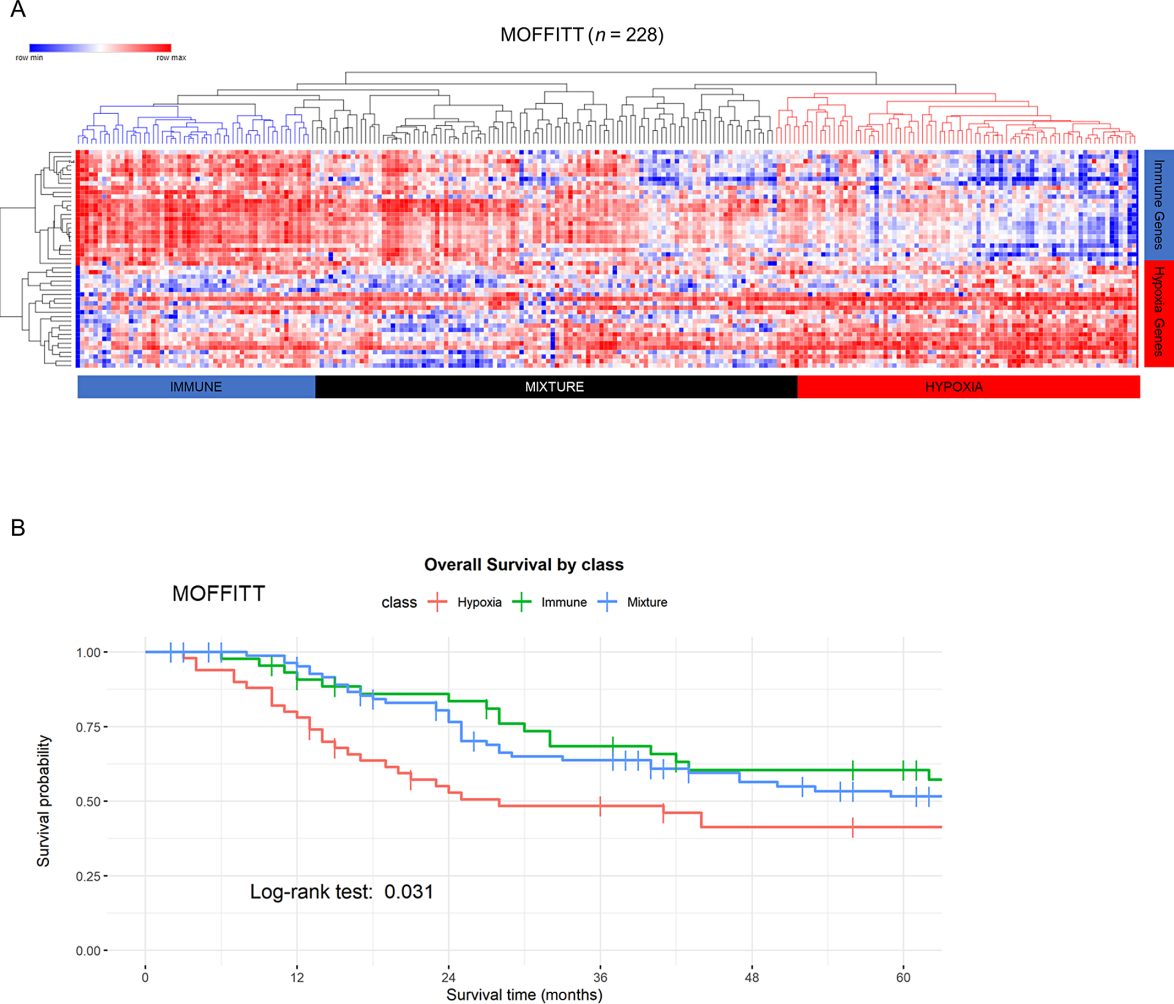
Molecular classification and validation of hypoxia-immune signature in HNSCC. **A,** Heatmap of molecular subgroups in the Moffitt (*n* = 228) HNSCC cohort. RNA expression data were z-normalized, each row represents a single gene in the hypoxia-immune signature gene list, each column represents a patient sample. Samples were reordered according to its molecular subgroup: Immune (blue), Mixture (black), and Hypoxia (red). **B,** Kaplan–Meier survival plots for overall survival stratified according to the Hypoxia-Immune signature for the Moffitt (*n* = 184) HNSCC patient cohort.

To identify hypoxic tumors reliably in our Moffitt HNSCC dataset for comprehensive characterization of TME, we assessed the Hypoxia-Immune signature (14) in three HNSCC datasets: TCGA (*n* = 519; Supplementary Table S1), CPTAC (*n* = 108; Supplementary Table S2; ref. 30), and a Moffitt cohort (*n* = 228; Table 1). Of the 54 genes from this signature, 49 were detectable in each of the three cohorts (Supplementary Table S3). On the basis of this 49-gene signature, TCGA tumors were assigned to one of three subgroups: Hypoxia (*n* = 168), Immune (*n* = 99), and Mixture (*n* = 252; Supplementary Fig. S1A), and CPTAC tumors were assigned to Hypoxia (*n* = 33), Immune (*n* = 26), and Mixture (*n* = 49; Supplementary Fig. S1B). The clustering pattern and proportions of the tumors assigned to the three subgroups were consistent with those in a previous publication (14). Similarly, the Moffitt cohort was analyzed by assigning the tumors to the Hypoxia (*n* = 76), Immune (*n* = 52), and Mixture (*n* = 100) subgroups (Fig. 1A). The clinical significance of hypoxic HNSCC as a poor prognostic biomarker was demonstrated by worse 5-year overall survival in the Hypoxia subgroup compared with the Immune and Mixture subgroups in TCGA (Supplementary Fig. S1C) and Moffitt cohorts (Fig. 1B), whereas the CPTAC follow-up information was too short for a meaningful survival analysis.

### Hypoxic HNSCC has Decreased Effector Lymphocytes and Increased Infiltration of Immunosuppressive Cells

To further determine tumor-infiltrating immune cell characteristics in the TME, we performed mIHC staining on 90 FFPE tissue samples on TMAs from the Moffitt cohort. Among these 90 tumors, the subgroup assignments were Hypoxia (*n* = 37), Immune (*n* = 15), and Mixture (*n* = 38). We measured the expression of 26 immune markers representing different lymphoid and myeloid lineages, along with CAIX, to assess hypoxia (see Materials and Methods for marker details).

**FIGURE 2:**
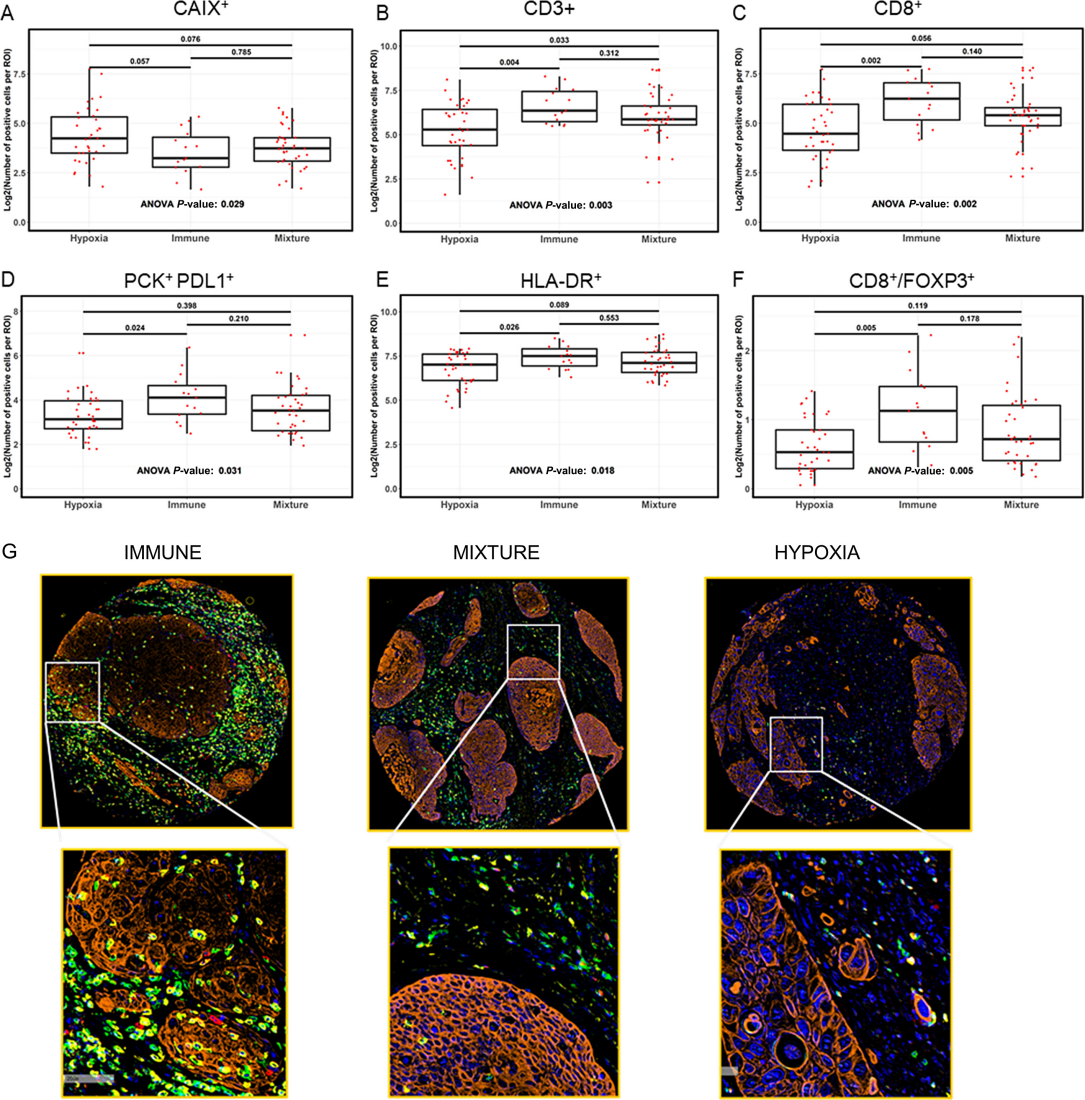
Hypoxia subgroup has immunosuppressive TME. Boxplots showing cell counts distribution of different immune markers CAIX^+^ cells representing hypoxia marker (**A**), CD3^+^ T cells (**B**), CD8^+^ cytotoxic T cells (**C**), PCK^+^ tumor cells expressing PD-L1 (**D**), HLA-DR^+^ APCs (**E**), and CD8^+^ T cells/FOXP3^+^ Tregs (**F**), among the three subgroups in the TMA mIHC cohort (*n* = 90). Statistical analysis was performed by ANOVA test and followed by Tukey *post hoc* tests. **G,** Representative mIHC images showing that the average numbers of immune cells expressing CD3^+^ T cells (green color), CD8^+^ cytotoxic T cells (yellow color), CD69^+^ activated T cells (red color), CD103^+^ resident memory T cells (cyan color) within 20 μm of a PCK^+^ cell (orange color) was statistically significantly lowest in the Hypoxia subgroup compared with the Mixture and Immune subgroups. Nuclei are shown in blue. Individual TMA core was considered as a region of interest (ROI). Bottom panel represents the zoom-in distance proximity and spatial distribution between a tumor cell and an immune cell within the TME.

The Hypoxia subgroup had significantly higher numbers of cells positive for the hypoxia marker CAIX than the other two subgroups (Fig. 2A). Effector lymphocytes were present in lower numbers in the Hypoxia subgroup than in the Immune and Mixture subgroups. These differences were statistically significant for several lymphocyte markers, including CD3^+^ T cells (Fig. 2B), CD8^+^ cytotoxic T cells (Fig. 2C), PD-L1–expressing PCK^+^ tumor cells (PCK^+^PD-L1^+^) (Fig. 2D), CD3^+^ CD4^+^ Th cells, CD69^+^ activated T cells, and CD103^+^ resident memory T cells (Supplementary Fig. S2A; Supplementary Table S4). We also evaluated proinflammatory M1 macrophages and found that HLA-DR^+^ antigen-presenting cells (APC; Fig. 2E) and CD163^−^HLA-DR^+^ M1 macrophages (Supplementary Fig. S2B; Supplementary Table S4) were significantly lower in the Hypoxia subgroup than in the other subgroups. Interestingly, the Mixture subgroup had a significantly higher number of APCs represented by MHCII^+^ cells and MHCII^+^HLA-DR^+^ cells than the Hypoxia subgroup (Supplementary Fig. S2B; Supplementary Table S4). In contrast to these proimmune cells, immunosuppressive cells were increased in the Hypoxia subgroup compared with those in the Immune and Mixture subgroups. The ratio of CD8^+^ T cells/FOXP3^+^ regulatory T cells (Treg) was significantly lower in the Hypoxia subgroup, suggesting immunosuppression by Tregs (Fig. 2F).

We evaluated the spatial distribution between a tumor cell (PCK^+^) and an immune cell by measuring the average cellular distances within the TME. Among the 90 FFPE samples mentioned above, we found that the average numbers of immune cells expressing CD3^+^ T cells, CD8^+^ cytotoxic T cells, CD69^+^ activated T cells, and CD103^+^ resident memory T cells within 20 μm of a PCK^+^ cell was significantly lower in the Hypoxia subgroup than in the Mixture and Immune subgroups (Fig. 2G; Supplementary Table S4). Fewer immune cells in the vicinity of tumor cells suggest that hypoxic tumors have a lower number of tumor-infiltrating lymphocytes (TIL) than tumors in the Immune and Mixture subgroups. Our results demonstrate that tumors in the Hypoxia subgroup have overall lower numbers of proinflammatory cells, including HLA-DR^+^ APCs and T lymphocytes, with less proximity to the tumor cells, suggesting a lack of trafficking of the immune cells toward the tumor cells, but higher numbers of immunosuppressive Tregs compared with the Immune and Mixture subgroups.

In addition, to determine the potential association among PD-L1 CPS and the Hypoxia, Immune, and Mixture subgroups, we determined PD-L1 CPS in 30 whole tumor slides from the Moffit cohort. For seven of 30 tumors, the PD-L1 staining was performed as a standard-of-care, Clinical Laboratory Improvement Amendments (CLIA)-certified assay, whereas the PD-L1 staining in the remaining 23 tumors was performed using a research mIHC protocol (see Materials and Methods for staining details). The 30 tumors with available PD-L1 CPS were assigned to each subgroup (Hypoxia *n* = 12, Immune *n* = 12, Mixture *n* = 6). The tumors in the Immune subgroup had higher PD-L1 CPS compared with other subgroups (full linear model *P* = 0.006; Supplementary Fig. S2C). Altogether, these features may contribute to a poor immunotherapy response for patients with hypoxic tumors.

### Recurrent Tumors Have Immunosuppressive TME as Compared with Newly Diagnosed HNSCC

We previously reported that recurrent HNSCCs have less tumor immune cell infiltration than primary HNSCCs (15). Therefore, we further examined the immune cell profiles in primary tumors (*n* = 63/90) and recurrent primary tumors (*n* = 20/90) among the 90 FFPE tissue samples available from the Moffitt cohort (Table 1). Although the primary and recurrent primary tumors were not paired tumor samples obtained longitudinally from a same patient, we found that primary tumors as a group had a significantly higher number of CD3^+^ T cells, MHCII^+^ APCs, and CD163^−^MHCII^+^ M1 macrophages than recurrent primary tumors as a group (Supplementary Fig. S2D), suggesting that recurrent tumors are more immunosuppressed than primary tumors. Interestingly, compared with newly diagnosed primary HNSCC, recurrent HNSCC had a significantly higher number of CD15^+^ cells and CD66b^+^ cells, both of which represent granulocyte activation and neutrophil lineage markers (Supplementary Fig. S2E). Furthermore, compared with primary tumors, recurrent tumors had significantly higher numbers of DC-SIGN^+^ cells (Supplementary Fig. S2E). Our data further indicate that compared with newly diagnosed primary HNSCC tumors, recurrent tumors have a more immunosuppressive TME.

We next evaluated whether hypoxia was associated with an immunosuppressive TME in recurrent tumors. Using the Hypoxia-Immune signature in all available Moffitt cohort samples (*n* = 228; Table 1), we found that 51/163 (31%) of newly diagnosed primary tumors and 22/49 (45%) of recurrent HNSCC were in the Hypoxia subgroup. To test whether recurrent tumors tend to be more hypoxic, we performed a trend test along the Hypoxia-Mixed-Immune spectrum and found that recurrent HNSCC tumors were more hypoxic than the primary tumors (Supplementary Table S5; Cochran–Armitage test: *P* = 0.048). Hypoxic TME in recurrent tumors may further contribute to an immunosuppressive TME.

### Clinical Benefits of Pembrolizumab/Nivolumab Among the Subgroups

To assess the correlation between hypoxia and response to immunotherapy, we retrospectively evaluated tumors from 48 patients treated with pembrolizumab or nivolumab monotherapy as a standard of care (MCC#18754). Using the Hypoxia-Immune signature, these tumors were classified into the same three subgroups. For 48 patients treated with at least two doses of pembrolizumab or nivolumab monotherapy, 16 were in Hypoxia, 14 were in Immune, and 18 were in Mixture (Supplementary Fig. S3). The patients with tumors belonging to the Hypoxia or Mixture subgroups had partial or incomplete disease control compared with those in the Immune subgroup (Table 2, *P* = 0.021, Cochran–Armitage test for trend). Although the retrospective evaluation of tumor response is a limitation, the data further support the evidence that single-agent immunotherapy is less likely to induce a clinical response in tumors belonging to the Hypoxia subgroup than in the Mixture or Immune subgroups.

### Signaling Pathways Associated with Hypoxia in HNSCC

**FIGURE 3:**
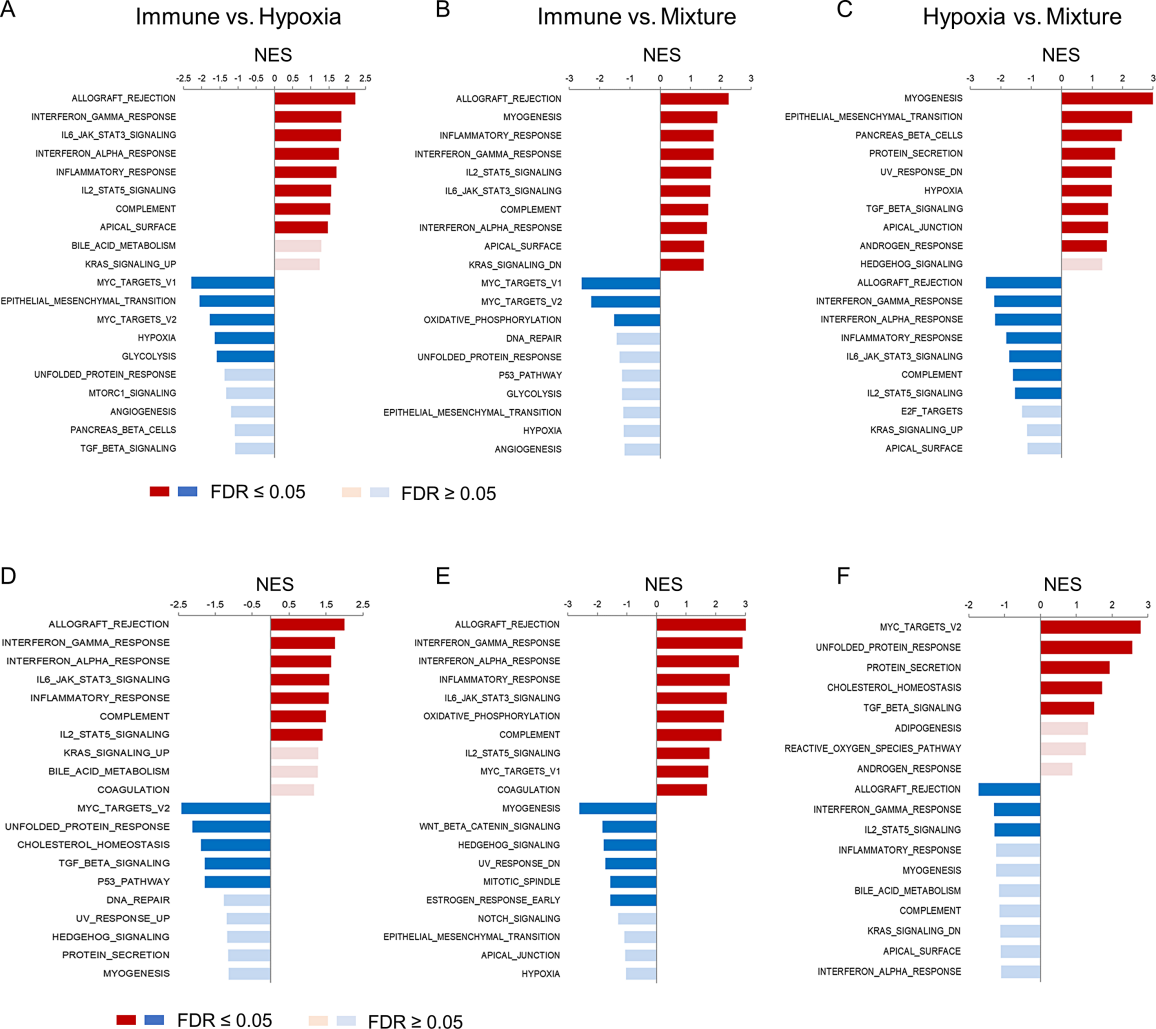
GSEA of hallmark pathways in TCGA and Moffitt cohorts. TCGA (**A–C**) and Moffitt head and neck squamous cell carcinoma (**D–F**) cohorts. The gene expressions were compared for pathways enriched in: (**A, D**) Immune versus Hypoxia. Red: enriched in Immune, Blue: enriched in Hypoxia. B and E, Immune versus Mixture. Red: enriched in Immune, Blue: enriched in Mixture. C and F, Hypoxia versus Mixture. Red: enriched in Hypoxia, Blue: enriched in Mixture. Indicated are the top 10 most differentially expressed pathways in Cancer Hallmarks gene sets. NES: Normalized enrichment score.

To gain a deeper understanding of the Hypoxia subgroup and to further investigate the potential therapeutic targets, we used GSEA on TCGA, CPTAC, and Moffitt HNSCC cohorts for Hallmarks of Cancer from the MSigDB. Using FDR < 0.05 as the cutoff, we found that, compared with the Hypoxia subgroup, the Immune subgroup was mostly enriched for proinflammatory pathways for IFNα and IFNγ responses, IL2-STAT and IL6-JAK-STAT3 signaling, general inflammatory responses, and others (Fig. 3; Supplementary Fig. S4). The Mixture subgroup was enriched in the E2F pathway and other pathways that control cellular growth (Fig. 3; Supplementary Fig. S4). As expected, the Hypoxia subgroup showed enrichment of hallmark hypoxia genes and decreased expression of IFNα and IFNγ response genes compared with the other subgroups. Interestingly, tumors in the Hypoxia subgroup were enriched for TGFβ signaling in both TCGA and Moffitt cohorts (Fig. 3; Supplementary Fig. S4). An increase in TGFβ signaling indicates a link with EGFR signaling, because TGFβ is known to transactivate EGFR (31). To further establish this connection, we performed a protein network analysis by taking core enriched genes within the Hypoxia signature (Supplementary Table S6) and TGFβ signaling from the GSEA (Supplementary Table S7) and clustered them using the STRING database (Supplementary Fig. S5). In this analysis, we observed three main clusters centered around the hypoxia, TGFβ, and EGFR signaling pathways, suggesting interactions among the three pathways.

**FIGURE 4:**
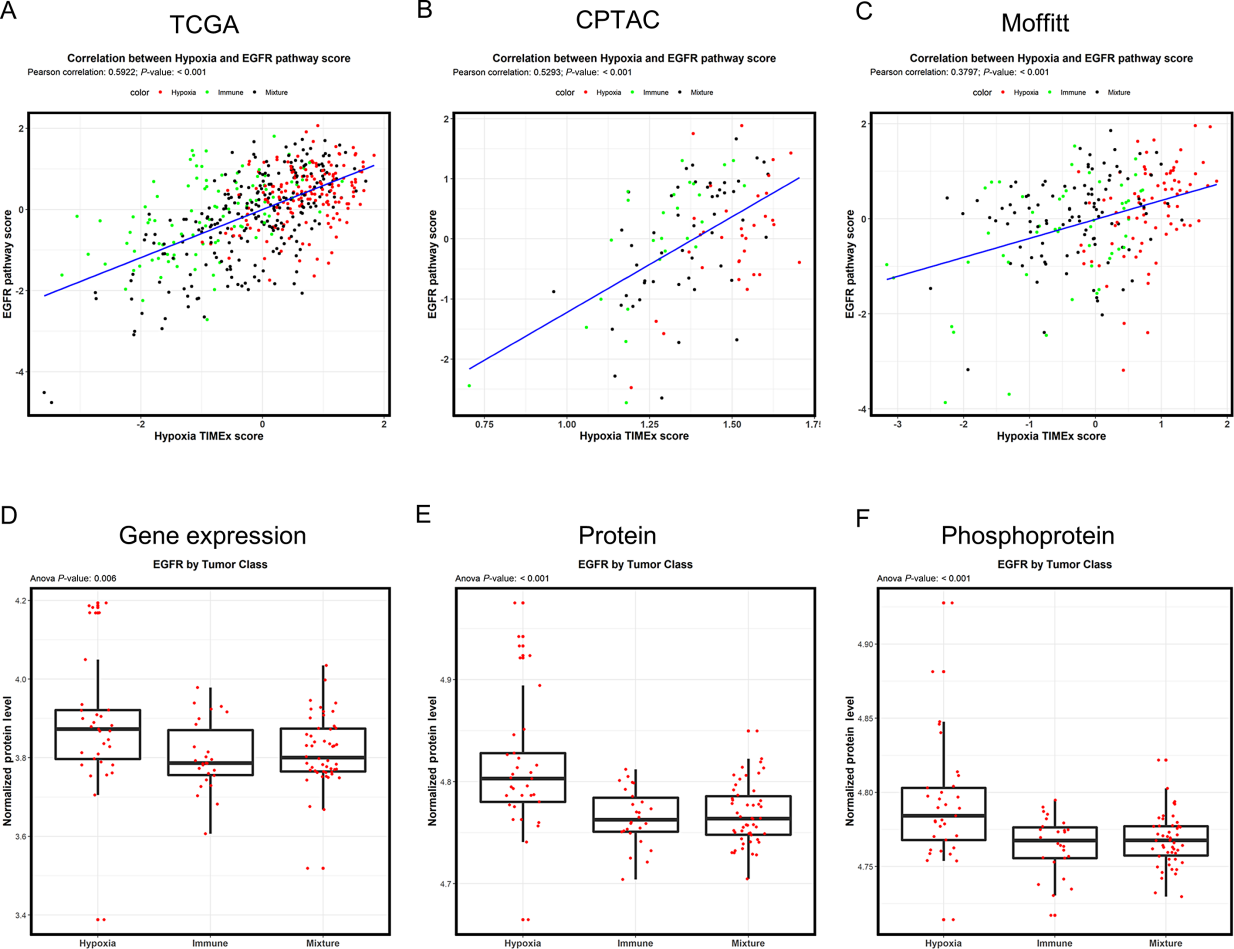
Hypoxia subgroup has an enrichment of EGFR pathway. Pearson correlation coefficient for the TIMEx scores for the EGFR pathway and the Hypoxia gene signature among the TCGA (**A**), CPTAC (**B**), and Moffitt (**C**) HNSCC datasets. Expression distribution of EGFR RNA (**D**), protein (**E**), and phosphoprotein (**F**) levels among the three subgroups in the CPTAC HNSCC cohort.

It is well established that HIF1α induces the expression of hypoxia-related genes and that HIF1α signaling can be activated by EGFR signaling (32, 33). STRING analysis revealed a connection between hypoxia and EGFR. Therefore, we further evaluated the correlation between TIMEx scores (29) for the EGFR pathway and the hypoxia gene signature among the three HNSCC datasets. We found a moderate but statistically significant correlation between the hypoxia gene signature and EGFR pathway scores in TCGA (Fig. 4A) and CPTAC (Fig. 4B) datasets (Pearson correlation of 0.59 and 0.52, respectively), and an intermediate but statistically significant correlation between the two scores in the Moffitt cohort (Pearson correlation = 0.38; Fig. 4C). Next, we queried the CPTAC gene, protein, and phosphoprotein datasets to evaluate EGFR expression levels among the three subgroups. Here, we found significantly higher EGFR expression levels in the Hypoxia subgroup than in the Immune and Mixture subgroups (Fig. 4D). Similarly, we found significantly higher EGFR protein (Fig. 4E) and phosphoprotein levels in the Hypoxia subgroup (Fig. 4F), indicating that EGFR signaling was significantly upregulated in the Hypoxia subgroup compared with the other two subgroups.

### EGFR Inhibition by Cetuximab Modulates Hypoxia and IFN Response Genes in the Hypoxia Subgroup

**FIGURE 5:**
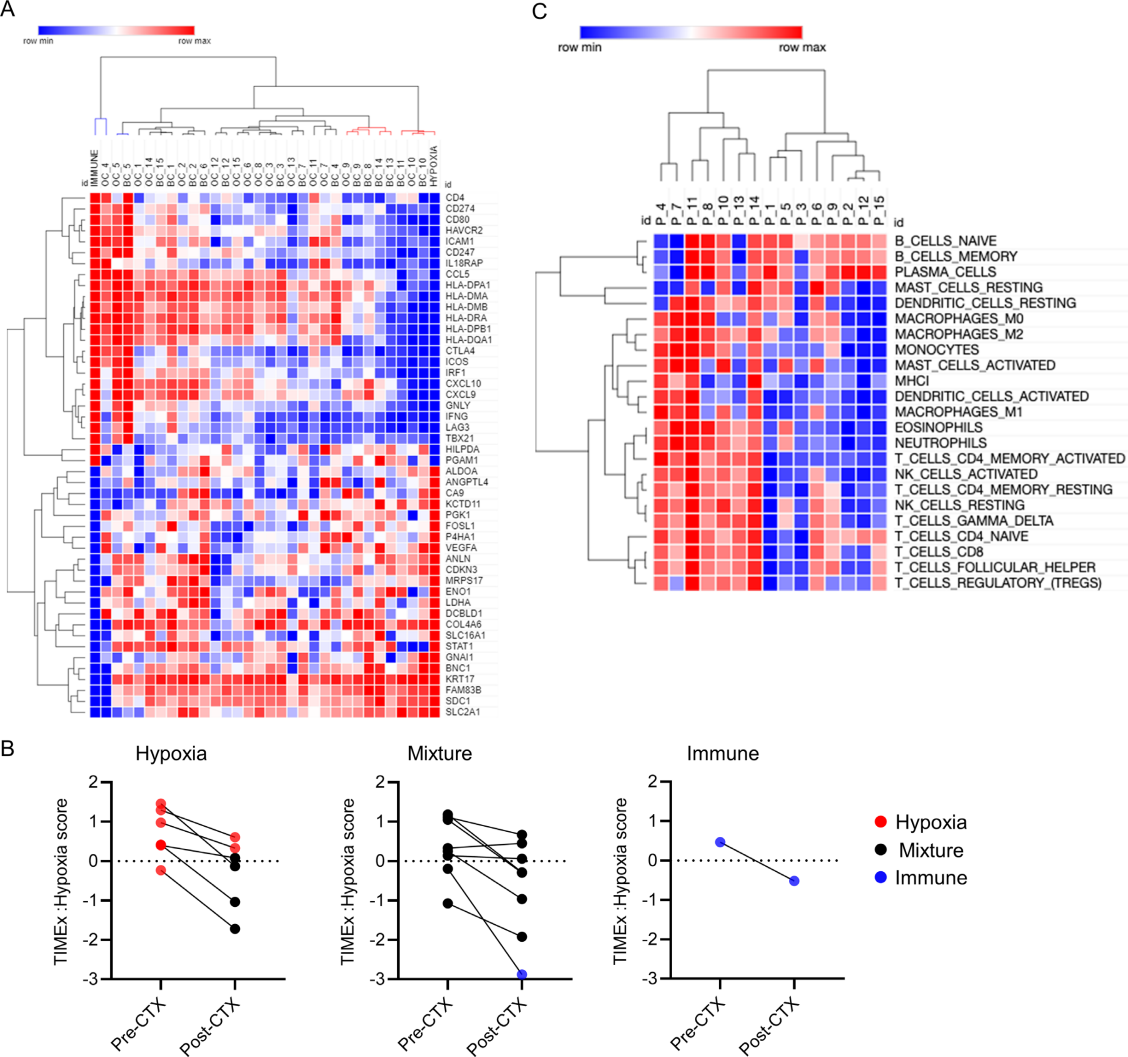
Cetuximab treatment benefits Hypoxia subgroup. **A,** Heatmap of gene expression data from pre- and post-cetuximab (CTX) treated patient samples. RNA expression data (GSE109756) was z-normalized, each row represents a single gene in the hypoxia-immune signature gene list, each column represents a patient sample. Hierarchical clustering was performed as detailed in Materials and Methods. Samples were reordered according to its molecular subgroup: Immune (blue), mixture (black), and Hypoxia (red) with pre-cetuximab treated samples labeled BC and post-cetuximab labeled OC. TIMEx scores for the Hypoxia gene signature among the 15 patients classified into the three subgroups (**B**) and GSEA comparing gene expression changes in the pre- and post-cetuximab data using CIBERSORT gene sets: higher in post-cetuximab (red) and higher in pre-ceuximab (blue) (**C**).

To assess the effects of EGFR inhibition on the genes in the Hypoxia-Immune signature, we evaluated pre- and post-cetuximab gene expression from a window-of-opportunity study in patients with HNSCC treated with three doses of cetuximab prior to surgery, as described previously (34). Using the Hypoxia-Immune signature, we classified the pre- and post-cetuximab treatment samples into the Hypoxia, Immune, and Mixture subgroups (Fig. 5A). Of the 15 baseline tumors, eight were classified as Mixture, six as Hypoxia, and one as Immune (Fig. 5A; Supplementary Table S8). Interestingly, after cetuximab treatment, the gene expression profiles of four of six Hypoxia subgroup tumors changed to the Mixture subgroup, and one of eight Mixture subgroup tumors changed to the Immune subgroup (Fig. 5A; Supplementary Table S8). This suggests a significant shift toward improvement of hypoxia given cetuximab treatment (*P* = 0.032, one-sided exact Wilcoxon–Pratt signed-rank test).

Next, we evaluated the expression of the immune gene cluster and hypoxia gene cluster in all pre- and post-cetuximab tumors. The immune TIMEx score increased in six of 15 tumors while the remaining nine tumors had either no change or decreased given cetuximab treatment (Supplementary Fig. S6). Interestingly, four of these six tumors with the increased immune TIMEx score belong to the Hypoxia subgroup although it was not statistically significant (*P* = 0.09, one-sided Student *t* test), considering the small sample size. On the other hand, there was a significant decrease in the hypoxia TIMEx score in all the tumors, except in one belonging to the Mixture subgroup (*P* < 0.001, one-sided Student *t* test; Fig. 5B), suggesting that cetuximab may reverse some of the effects of hypoxia in TME in HNSCC. These results are intriguing and further support our previous observation that tumors in the Hypoxia subgroup may benefit from cetuximab treatment by reducing hypoxia in the TME. To further validate this observation, we evaluated changes in the expression of immune cells in response to cetuximab treatment. We used CIBERSORT gene signatures and performed GSEA to compare immune cell changes between pre- and post-cetuximab tumors. We identified a statistically significant increase in T-cell and B-cell populations after cetuximab treatment (Fig. 5C). These changes were more significant in the left branch of the cluster, mostly representing tumors from the Hypoxia subgroup.

## Discussion

HNSCC is a heterogeneous disease with respect to biology and clinical behavior. The lack of meaningful prognostic and predictive biomarkers beyond HPV status and limited treatment options highlights the need to better understand the biology of HNSCC. Substantial progress has been made over the past decade in the molecular characterization of HNSCC (35–37). Recently, Brooks and colleagues identified a prognostic molecular classifier using a 54-gene Hypoxia-Immune signature based on data available in literature (14). Hypoxia is a common feature in solid tumors, including HNSCC, and contributes to tumor development and metastasis. Within the TME, hypoxia is associated with poor clinical outcomes, increased treatment resistance, increased tumor heterogeneity, and decreased overall patient survival (38). Hypoxic HPV-negative HNSCC tumors benefit from the use of hypoxia modifiers such as nimorazole compared with HPV-positive tumors (39). Therefore, in this study, we focused only on HPV-negative tumors for subsequent analysis of our in-house Moffitt cohort.

Hypoxia induces immunosuppressive TME (40). In our study, tumors in the Hypoxia subgroup had decreased numbers of T lymphocytes, APCs (HLA-DR^+^ or MHCII^+^ cells), and proinflammatory CD163^−^HLA-DR^+^ M1 macrophages, as well as a significant decrease in the ratio of cytotoxic T cells/FOXP3^+^ Tregs as compared with the Immune and Mixture subgroups. Next, we found recurrent tumors had significantly higher number of CD15^+^ cells and CD66b^+^ cells representing neutrophils and DC-SIGN^+^ cells, as compared with primary tumors. Neutrophils exhibit functional plasticity and can exert protumor or antitumor effects in a context-dependent manner (41). DC-SIGN^+^ protein can be expressed on both macrophages and dendritic cells (DC), and DC-SIGN^+^ tumor-associated macrophages have immunosuppressive and tumor-promoting functions (42). Furthermore, we found that a greater percentage of recurrent tumors belonged to the Hypoxia subgroup with an immunosuppressive TME compared with newly diagnosed primary HNSCC.

Hypoxia-driven immunosuppression can occur through multiple mechanisms, many of which are HIF1α-mediated (43). Hypoxia reduces the activation levels of TILs, recruitment of suppressive chemokines and cytokines, and activation of immunosuppressive cells such as Tregs and MDSCs, resulting in immunosuppression and evasion of immune detection (38, 44). It is well established that for the effective killing of cancer cells, all sequential steps of the cancer-immunity cycle have to be intact (45). Our data suggest that hypoxic tumors may have defects in the early stages of this cycle owing to the lack of appropriate antigen presentation and T-cell priming, activation, and trafficking. These defects might translate into a lack of response to immune checkpoint inhibitors, such as anti-PD-1 inhibitors (e.g., pembrolizumab or nivolumab), which exert their therapeutic effect at the later steps of the cycle reinvigorating exhausted T cells already present in the TME. In our exploratory retrospective analyses of tumors from patients who were treated with pembrolizumab or nivolumab monotherapy as standard care, fewer tumors belonging to the Hypoxia subgroup had a favorable clinical response compared with those in the Immune subgroup. This further indicates that tumors belonging to the Immune subgroup will respond better to the anti-PD-L1 immunotherapy compared with the Hypoxia and Mixture subgroups.

Prior clinical trials of hypoxia-targeting agents combined with chemotherapy and/or radiation for the treatment of HNSCC did not show clinical benefits (46). In this study, we attempted to identify signaling pathways that could be exploited to target hypoxic tumors and restore the proinflammatory TME. We performed GSEA and identified enrichment of TGFβ signaling pathway and downregulation of IFN signaling in the Hypoxia subgroup. TGFβ signaling can suppress immune responses by inhibiting the function of inflammatory cells and promoting the expansion of Tregs (47). While TGFβ inhibitors are currently not used in the standard-of-care cancer treatment (48), there are numerous EGFR inhibitors used in cancer care, including the anti-EGFR mAb, cetuximab, which is FDA approved for the management of HNSCC (49). Therefore, we focused our analyses on the EGFR pathway. IFNα administration enhances the antitumor effects of EGFR in HNSCC cells (50). However, it is unknown how EGFR signaling is linked to immune regulatory pathways in HNSCC. EGFR is overexpressed in 90% of patients with HNSCC with coexpression of ligands, predominantly TGFα and amphiregulin (51). EGFR inhibition induces apoptosis and inhibits cellular proliferation and invasion (52). Cetuximab competitively inhibits ligand binding (53). The response rate to cetuximab monotherapy is only 13% in patients with HNSCC (54). Nonetheless, the combination of cetuximab with other treatment regimens or targeted agents has shown better outcomes (55).

Our pathway analysis indicated that EGFR inhibitors, such as cetuximab, may have immune-enhancing therapeutic effects in hypoxic tumors. Prior research has shown that EGFR signaling pathway inhibitors can remodel the TME by inducing antibody-dependent cell-mediated cytotoxicity by activating natural killer cells and modulating T-cell priming by activating DCs (56, 57). In HNSCC, EGFR inhibition induces IFN pathway signaling and subsequent remodeling of the TME by increasing the expression of proinflammatory (CXCL10) and/or immunosuppressive (IL6) chemokines and cytokines (58). In recently published phase II clinical trial studies, combinations of the anti-PD-1 inhibitors pembrolizumab or nivolumab with cetuximab showed an overall response rate range of 23%–45% by 6 months, depending on the number of prior treatments in patients with recurrent or metastatic HNSCC (59–61). This study corroborates our hypothesis that patients with HNSCC with hypoxic tumors may benefit the most from a combination of cetuximab and an immune checkpoint blockade.

However, we acknowledge several limitations of our study. The association between the patients with hypoxic tumors and low PD-L1 CPS is based on a combined result of two different anti-PD-L1 antibodies using a CLIA-certified and a research mIHC protocols, introducing a technical variability in the staining quality and quantity. The association between the patients with hypoxic tumors and poor response to current anti-PD-1 inhibitors is based on a retrospective dataset with a limited sample size. The data demonstrating modulation of TME by cetuximab by comparing pre- and post-cetuximab treatment was also obtained from bioinformatics analyses of a bulk tumor gene expression dataset with a limited sample size of only 15 paired tumors (34). These hypothesis generating analyses suggesting involvement of TGFβ and IFN signaling through EGFR pathway activation in hypoxic tumors and potential modulation of these pathways by cetuximab must be validated through further mechanistic evaluations using appropriate model systems as well as prospective studies in selected patients with hypoxic tumors.

In conclusion, our study demonstrates that a significant number of HNSCC have hypoxic TME and are immunosuppressed. The current therapeutic approach for anti-PD-1 inhibition may not be effective in patients with hypoxic tumors. However, remodeling the TME by targeting hypoxia-induced signaling pathways may potentially affect patient outcomes in HNSCC. We demonstrated that EGFR inhibition using cetuximab may play a role as a combination partner with immunotherapeutic agents in selected patients with hypoxic tumors.

## Supplementary Material

Supplementary Table S1 Supplementary Table S1. TCGA Cohort

Supplementary Table S2 Supplementary Table S2. CPTAC Cohort

Supplementary Table S3 Supplementary Table S3. 49-gene hypoxia-immune signature

Supplementary Table S4 Supplementary Table S4. TME across the Immune, Mixture and Hypoxia subgroups

Supplementary Table S5 Supplementary Table S5. Immune profile of the primary and recurrent tumors in Moffitt HNSCC cohort

Supplementary Table S6 Supplementary Table S6. Core enriched genes within the Hypoxia signature

Supplementary Table S7 Supplementary Table S7. Core enriched genes in the TGF-β signaling

Supplementary Table S8 Supplementary Table S8. Hypoxia-Immune classification of Schmitz et al., window-of-opportunity trial (GSE109756)

Supplementary Figure S1 (A, B) Heatmap of molecular subgroups in two different HNSCC datasets (A) TCGA and (B) CPTAC. RNA expression data was z-normalized, each row represents a single gene in the hypoxia-immune signature gene list, each column represents a patient sample. Samples were reordered according to its molecular subgroup: Immune (blue), Mixture (black) and Hypoxia (red). (C) Kaplan–Meier survival plots for overall survival stratified according to the hypoxia-immune signature for TCGA (n = 518) HNSCC patient cohorts.

Supplementary Figure S2 (A, B) Representative mIHC images showing staining of nuclei (blue), tumor cell (orange - PCK) and selected immune checkpoint markers in each molecular subgroup. (A) Images show four lymphoid markers - CD3+ T-cells (Green), CD8+ cytotoxic T-cells (Yellow), CD69+ activated T-cells (Red) and CD103+ resident memory T-cells (Cyan). (B) Images show three myeloid markers – HLA-DR+ APCs (Green), MHCII+ APCs (Cyan) and CD163+ M2 macrophages (Yellow). (C) Linear model showing correlation between PD-L1 CPS and Immune, Mixture and Hypoxia subgroups. (D-E) Boxplots showing cell counts distribution of different immune markers among the primary (n = 63) and recurrent (n = 20) tumors in TMA mIHC cohort. Students t-tests were used to test for significant differences between the groups.

Supplementary Figure S3 Heatmap of molecular subgroups in MCC18754 (n=48). RNA expression data was z-normalized, each row represents a single gene in the Hypoxia-Immune signature gene list, each column represents a patient sample. Samples were reordered according to its molecular subgroup: Immune (blue), Mixture (black) and Hypoxia (red).

Supplementary Figure S4 (A-C) Clinical Proteomic Tumor Analysis Consortium head and neck squamous cell carcinoma cohort. The gene expressions were compared for pathways enriched in: (A) Immune vs Hypoxia. Red: enriched in Immune, Blue: enriched in Hypoxia. (B) Immune vs Mixture. Red: enriched in Immune, Blue: enriched in Mixture. (C) Hypoxia vs Mixture. Red: enriched in Hypoxia, Blue: enriched in Mixture. Indicated are the top 10 most differentially expressed pathways in Cancer Hallmarks gene sets. NES: Normalized Enrichment Score

Supplementary Figure S5 Protein network analysis of core-enriched genes within the hypoxia signature and TGF-β signaling from GSEA analysis. Clustering was performed using the STRING database. Three main clusters are depicted: hypoxia (blue color), TGF-β (green color), and EGFR (red color).

Supplementary Figure S6 TIMEx scores for the Immune gene signature among the 15 patients classified into the Hypoxia, Mixture and Immune subgroups.
