# Supplementary material for: EGFR Inhibition by Cetuximab Modulates Hypoxia and IFN Response Genes in Head and Neck Squamous Cell Carcinoma
Source: Cancer Res Commun. 2023 May 22;3(5):896–907. doi: 10.1158/2767-9764.CRC-22-0443 (PMC10202124; doi:10.1158/2767-9764.CRC-22-0443)
Supplement: Supplementary Figure S1 — (A, B) Heatmap of molecular subgroups in two different HNSCC datasets (A) TCGA and (B) CPTAC. RNA expression data was z-normalized, each row represents a single gene in the hypoxia-immune signature gene list, each column represents a patient sample. Samples were reordered according to its molecular subgroup: Immune (blue), Mixture (black) and Hypoxia (red). (C) Kaplan–Meier survival plots for overall survival stratified according to the hypoxia-immune signature for TCGA (n = 518) HNSCC patient cohorts. [file crc-22-0443-s09.pptx]

## Slide 1
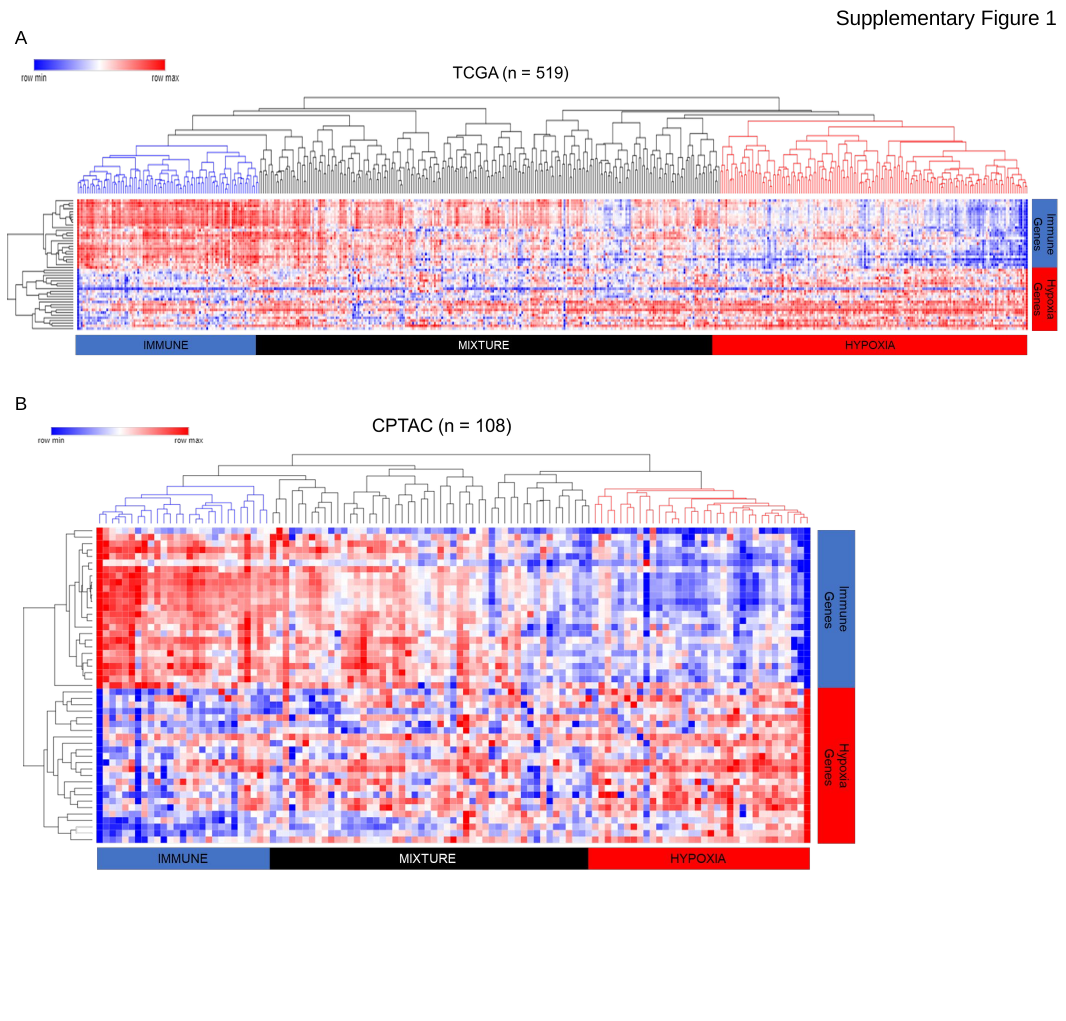

Supplementary Figure 1
A
B

## Slide 2
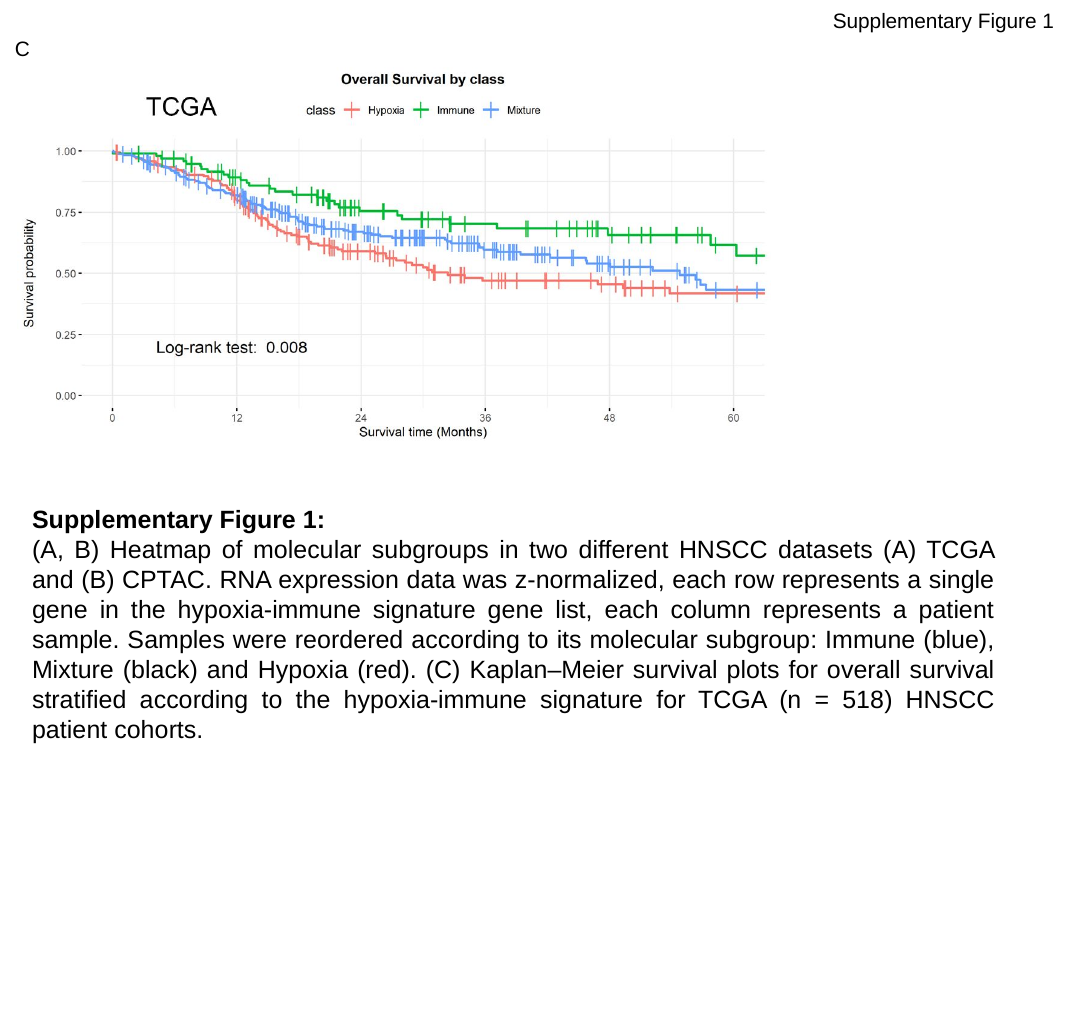

Supplementary Figure 1
C
Supplementary Figure 1:
(A, B) Heatmap of molecular subgroups in two different HNSCC datasets (A) TCGA and (B) CPTAC. RNA expression data was z-normalized, each row represents a single gene in the hypoxia-immune signature gene list, each column represents a patient sample. Samples were reordered according to its molecular subgroup: Immune (blue), Mixture (black) and Hypoxia (red). (C) Kaplan–Meier survival plots for overall survival stratified according to the hypoxia-immune signature for TCGA (n = 518) HNSCC patient cohorts.
