# Supplementary material for: EGFR Inhibition by Cetuximab Modulates Hypoxia and IFN Response Genes in Head and Neck Squamous Cell Carcinoma
Source: Cancer Res Commun. 2023 May 22;3(5):896–907. doi: 10.1158/2767-9764.CRC-22-0443 (PMC10202124; doi:10.1158/2767-9764.CRC-22-0443)
Supplement: Supplementary Figure S2 — (A, B) Representative mIHC images showing staining of nuclei (blue), tumor cell (orange - PCK) and selected immune checkpoint markers in each molecular subgroup. (A) Images show four lymphoid markers - CD3+ T-cells (Green), CD8+ cytotoxic T-cells (Yellow), CD69+ activated T-cells (Red) and CD103+ resident memory T-cells (Cyan). (B) Images show three myeloid markers – HLA-DR+ APCs (Green), MHCII+ APCs (Cyan) and CD163+ M2 macrophages (Yellow). (C) Linear model showing correlation between PD-L1 CPS and Immune, Mixture and Hypoxia subgroups. (D-E) Boxplots showing cell counts distribution of different immune markers among the primary (n = 63) and recurrent (n = 20) tumors in TMA mIHC cohort. Students t-tests were used to test for significant differences between the groups. [file crc-22-0443-s10.pptx]

## Slide 1
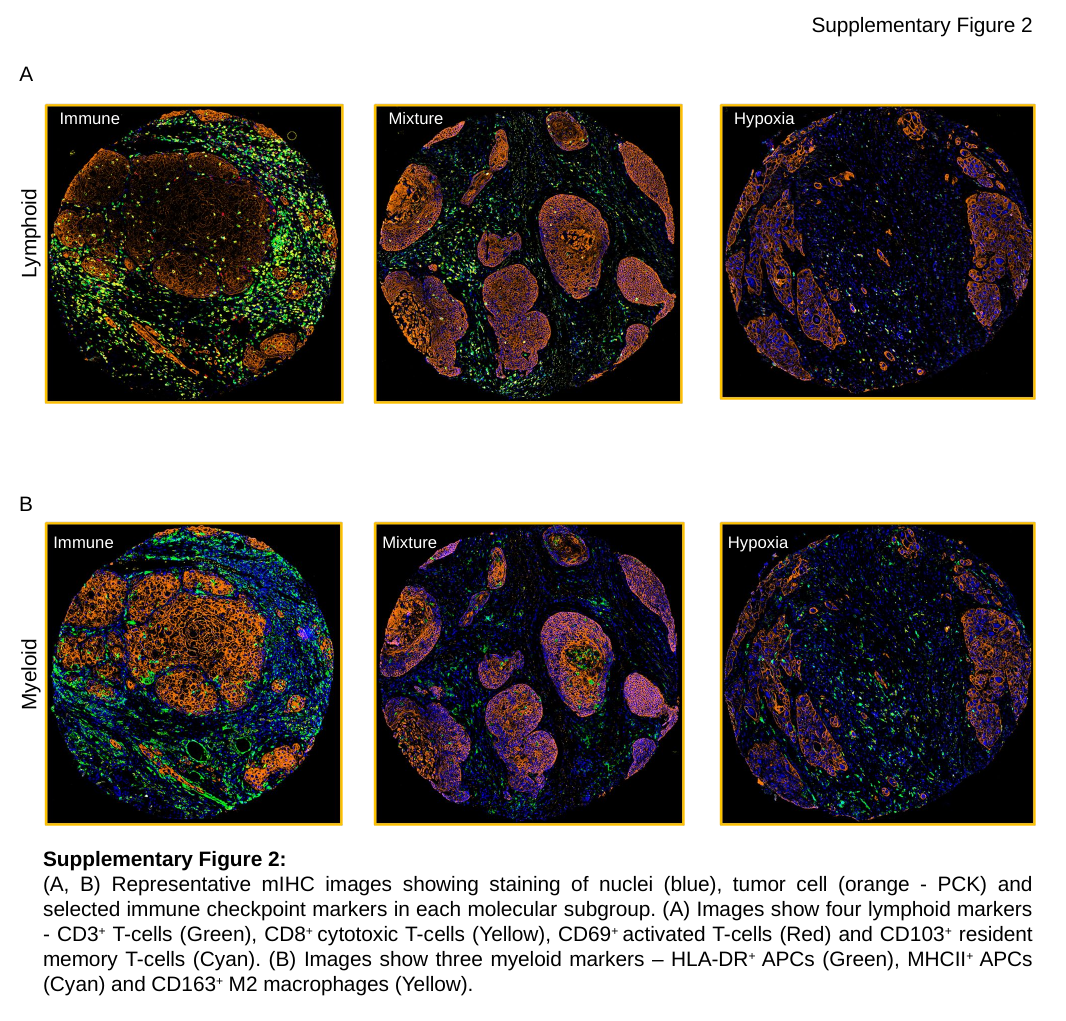

Supplementary Figure 2
A
Immune
Mixture
Hypoxia
Lymphoid
B
Immune
Mixture
Hypoxia
Myeloid
Supplementary Figure 2:
(A, B) Representative mIHC images showing staining of nuclei (blue), tumor cell (orange - PCK) and selected immune checkpoint markers in each molecular subgroup. (A) Images show four lymphoid markers - CD3+ T-cells (Green), CD8+ cytotoxic T-cells (Yellow), CD69+ activated T-cells (Red) and CD103+ resident memory T-cells (Cyan). (B) Images show three myeloid markers – HLA-DR+ APCs (Green), MHCII+ APCs (Cyan) and CD163+ M2 macrophages (Yellow).

## Slide 2
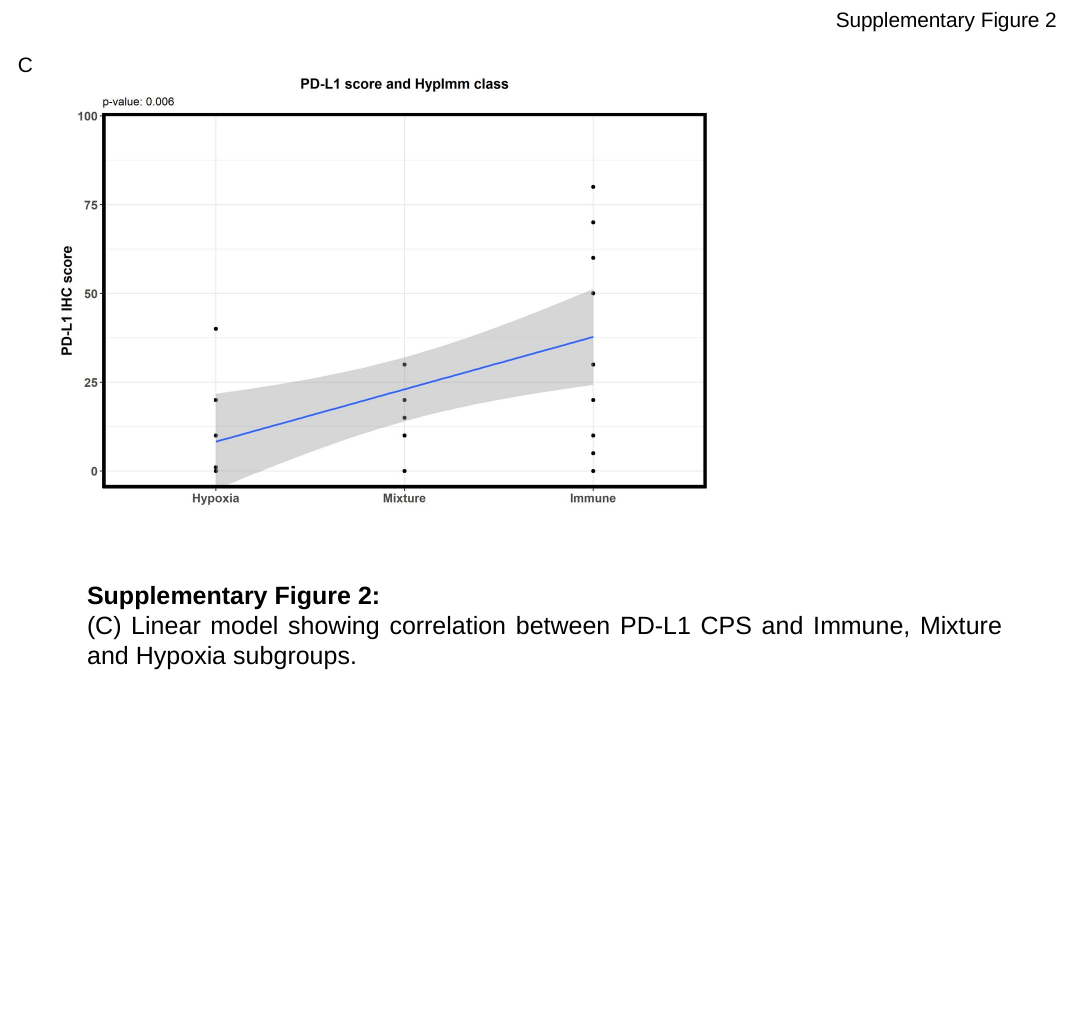

Supplementary Figure 2
C
Supplementary Figure 2:
(C) Linear model showing correlation between PD-L1 CPS and Immune, Mixture and Hypoxia subgroups.

## Slide 3
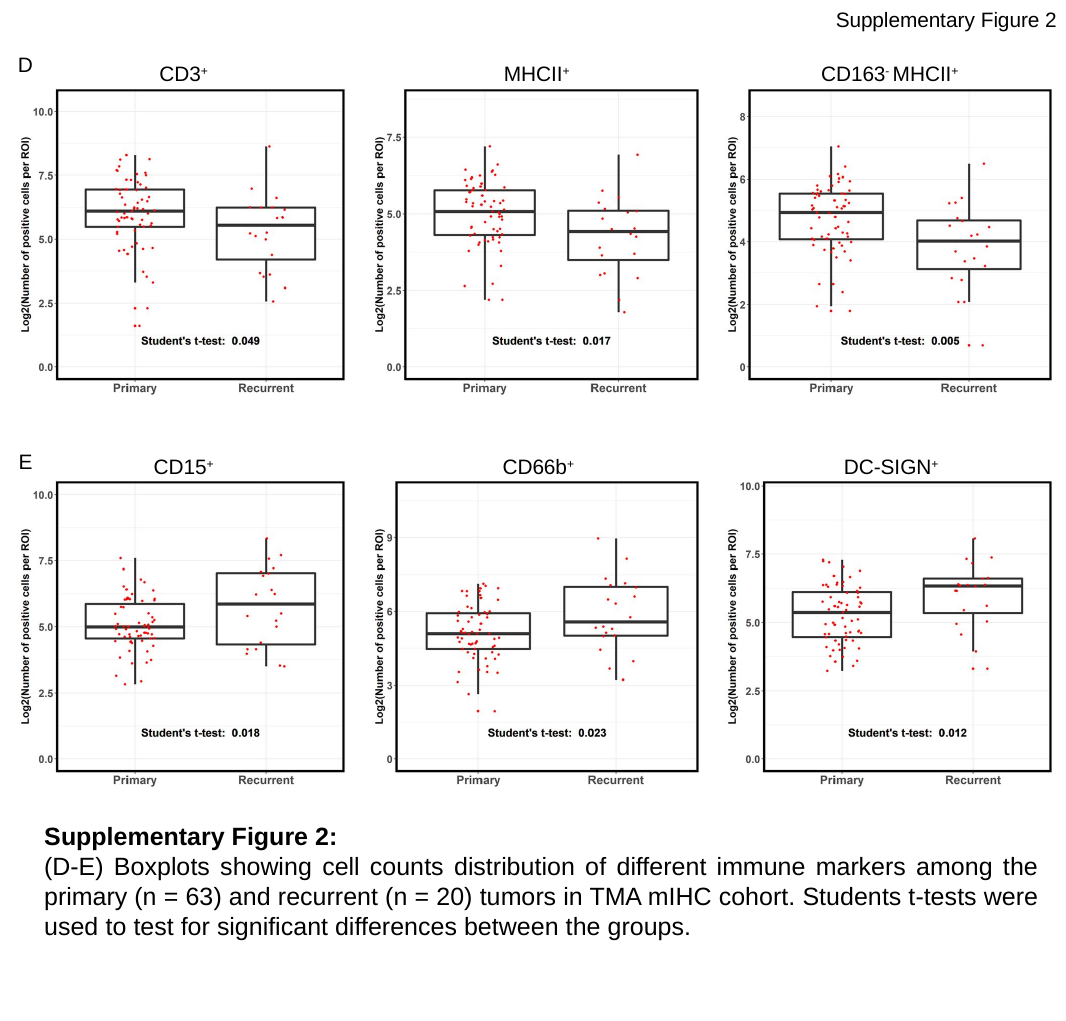

Supplementary Figure 2
D
CD3+
MHCII+
CD163- MHCII+
E
CD15+
CD66b+
DC-SIGN+
Supplementary Figure 2:
(D-E) Boxplots showing cell counts distribution of different immune markers among the primary (n = 63) and recurrent (n = 20) tumors in TMA mIHC cohort. Students t-tests were used to test for significant differences between the groups.
