# Supplementary material for: EGFR Inhibition by Cetuximab Modulates Hypoxia and IFN Response Genes in Head and Neck Squamous Cell Carcinoma
Source: Cancer Res Commun. 2023 May 22;3(5):896–907. doi: 10.1158/2767-9764.CRC-22-0443 (PMC10202124; doi:10.1158/2767-9764.CRC-22-0443)
Supplement: Supplementary Figure S3 — Heatmap of molecular subgroups in MCC18754 (n=48). RNA expression data was z-normalized, each row represents a single gene in the Hypoxia-Immune signature gene list, each column represents a patient sample. Samples were reordered according to its molecular subgroup: Immune (blue), Mixture (black) and Hypoxia (red). [file crc-22-0443-s11.pptx]

## Slide 1
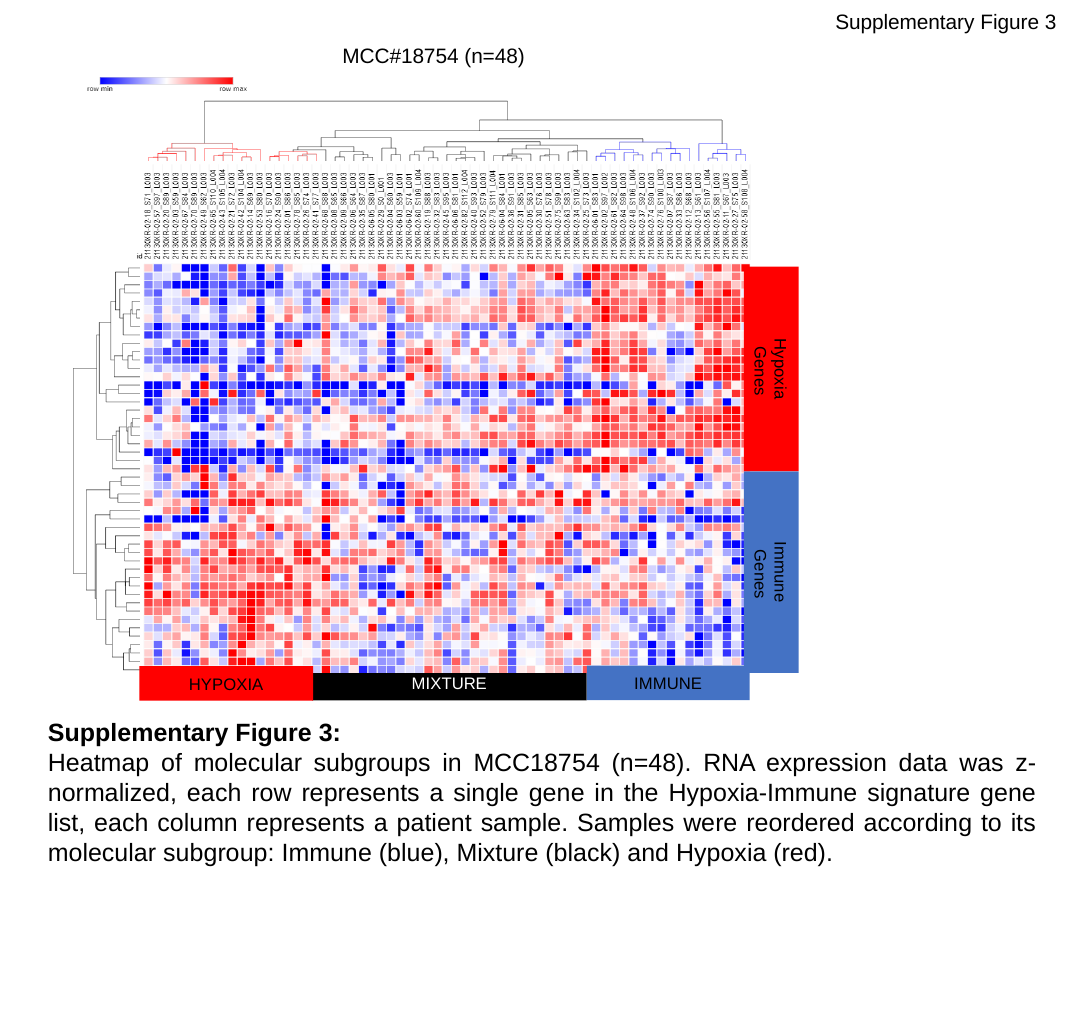

Supplementary Figure 3
 MCC#18754 (n=48)
Hypoxia
 Genes
Immune
 Genes
IMMUNE
MIXTURE
HYPOXIA
Supplementary Figure 3:
Heatmap of molecular subgroups in MCC18754 (n=48). RNA expression data was z-normalized, each row represents a single gene in the Hypoxia-Immune signature gene list, each column represents a patient sample. Samples were reordered according to its molecular subgroup: Immune (blue), Mixture (black) and Hypoxia (red).
