# Supplementary material for: EGFR Inhibition by Cetuximab Modulates Hypoxia and IFN Response Genes in Head and Neck Squamous Cell Carcinoma
Source: Cancer Res Commun. 2023 May 22;3(5):896–907. doi: 10.1158/2767-9764.CRC-22-0443 (PMC10202124; doi:10.1158/2767-9764.CRC-22-0443)
Supplement: Supplementary Figure S4 — (A-C) Clinical Proteomic Tumor Analysis Consortium head and neck squamous cell carcinoma cohort. The gene expressions were compared for pathways enriched in: (A) Immune vs Hypoxia. Red: enriched in Immune, Blue: enriched in Hypoxia. (B) Immune vs Mixture. Red: enriched in Immune, Blue: enriched in Mixture. (C) Hypoxia vs Mixture. Red: enriched in Hypoxia, Blue: enriched in Mixture. Indicated are the top 10 most differentially expressed pathways in Cancer Hallmarks gene sets. NES: Normalized Enrichment Score [file crc-22-0443-s12.pptx]

## Slide 1
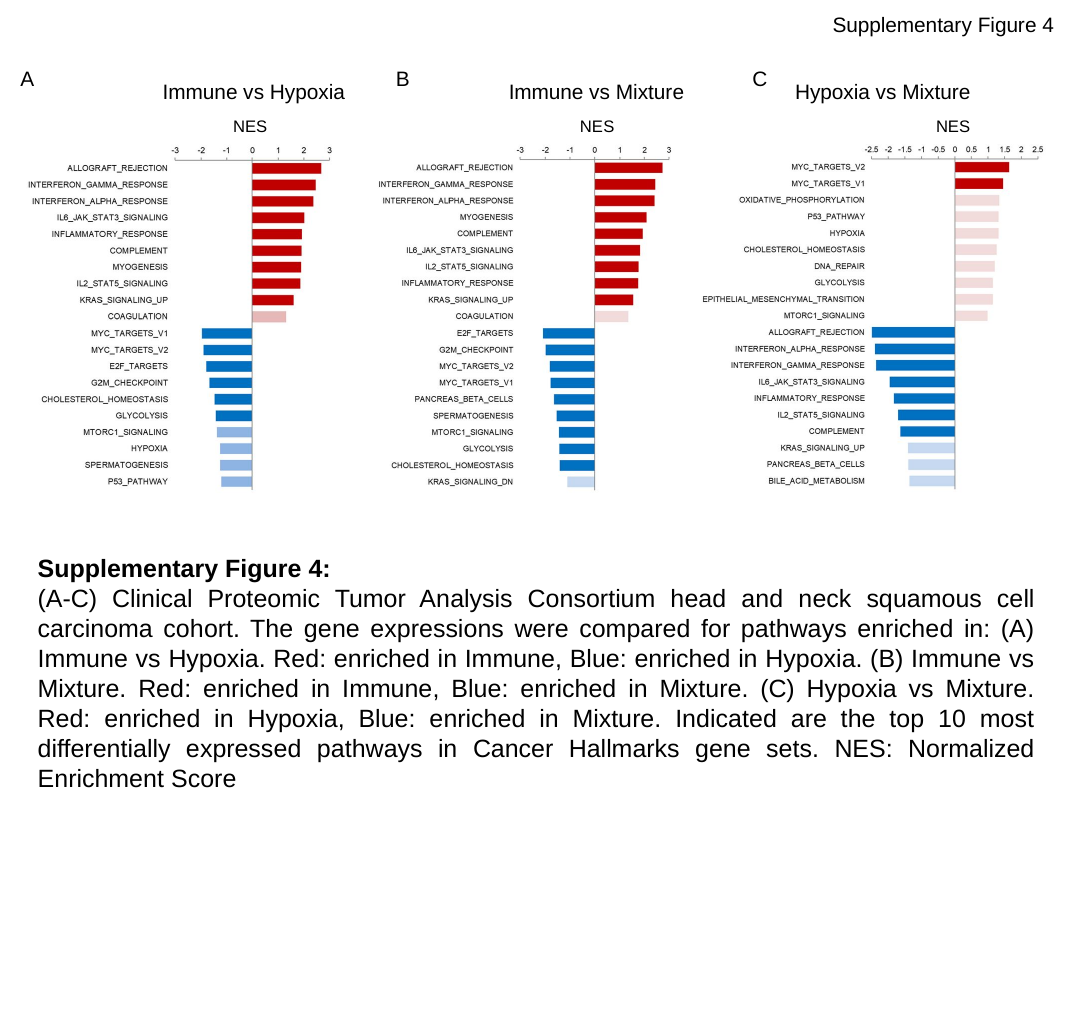

Supplementary Figure 4
A
B
C
Immune vs Hypoxia
Immune vs Mixture
Hypoxia vs Mixture
NES
NES
NES
Supplementary Figure 4:
(A-C) Clinical Proteomic Tumor Analysis Consortium head and neck squamous cell carcinoma cohort. The gene expressions were compared for pathways enriched in: (A) Immune vs Hypoxia. Red: enriched in Immune, Blue: enriched in Hypoxia. (B) Immune vs Mixture. Red: enriched in Immune, Blue: enriched in Mixture. (C) Hypoxia vs Mixture. Red: enriched in Hypoxia, Blue: enriched in Mixture. Indicated are the top 10 most differentially expressed pathways in Cancer Hallmarks gene sets. NES: Normalized Enrichment Score
