# Supplementary material for: EGFR Inhibition by Cetuximab Modulates Hypoxia and IFN Response Genes in Head and Neck Squamous Cell Carcinoma
Source: Cancer Res Commun. 2023 May 22;3(5):896–907. doi: 10.1158/2767-9764.CRC-22-0443 (PMC10202124; doi:10.1158/2767-9764.CRC-22-0443)
Supplement: Supplementary Figure S5 — Protein network analysis of core-enriched genes within the hypoxia signature and TGF-β signaling from GSEA analysis. Clustering was performed using the STRING database. Three main clusters are depicted: hypoxia (blue color), TGF-β (green color), and EGFR (red color). [file crc-22-0443-s13.pptx]

## Slide 1
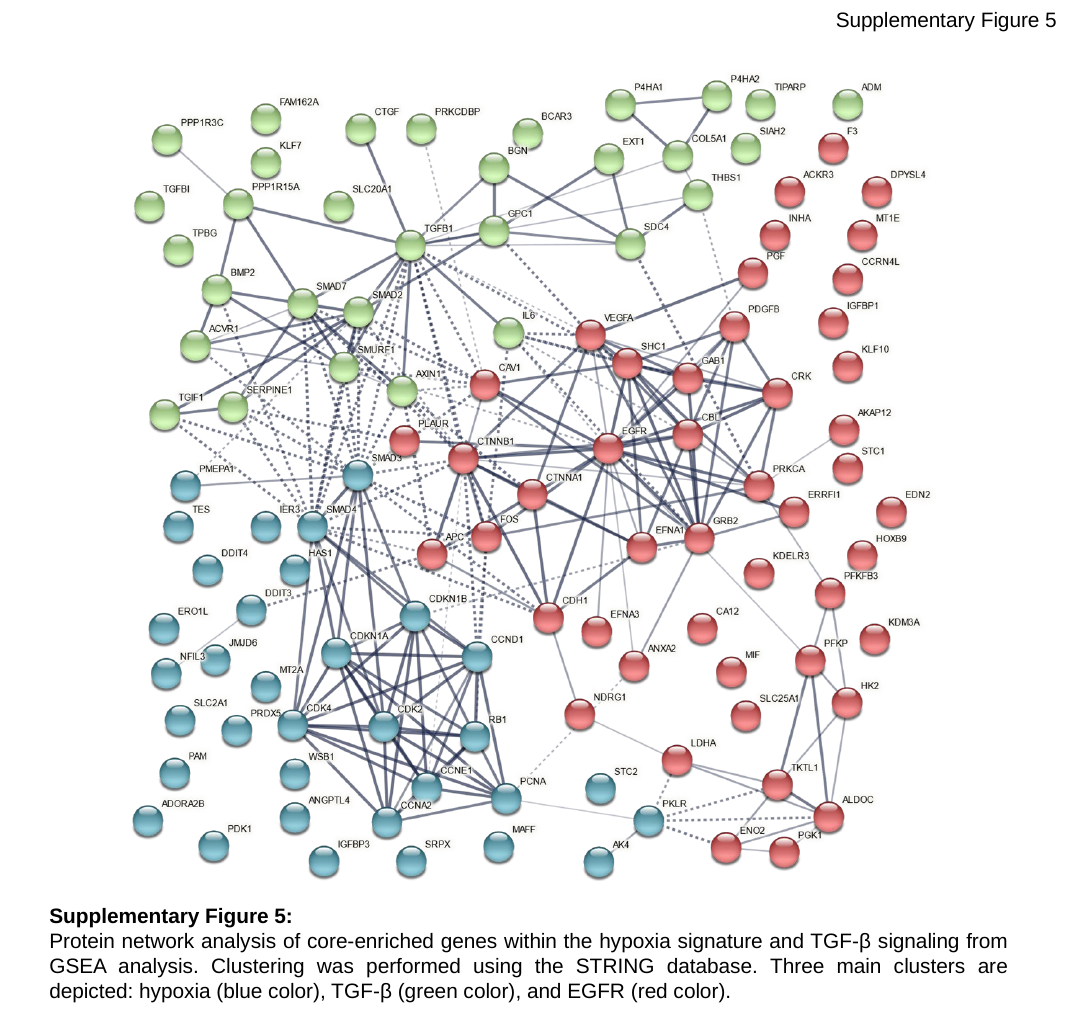

Supplementary Figure 5
Supplementary Figure 5:
Protein network analysis of core-enriched genes within the hypoxia signature and TGF-β signaling from GSEA analysis. Clustering was performed using the STRING database. Three main clusters are depicted: hypoxia (blue color), TGF-β (green color), and EGFR (red color).
