# Supplementary material for: EGFR Inhibition by Cetuximab Modulates Hypoxia and IFN Response Genes in Head and Neck Squamous Cell Carcinoma
Source: Cancer Res Commun. 2023 May 22;3(5):896–907. doi: 10.1158/2767-9764.CRC-22-0443 (PMC10202124; doi:10.1158/2767-9764.CRC-22-0443)
Supplement: Supplementary Figure S6 — TIMEx scores for the Immune gene signature among the 15 patients classified into the Hypoxia, Mixture and Immune subgroups. [file crc-22-0443-s14.pptx]

## Slide 1
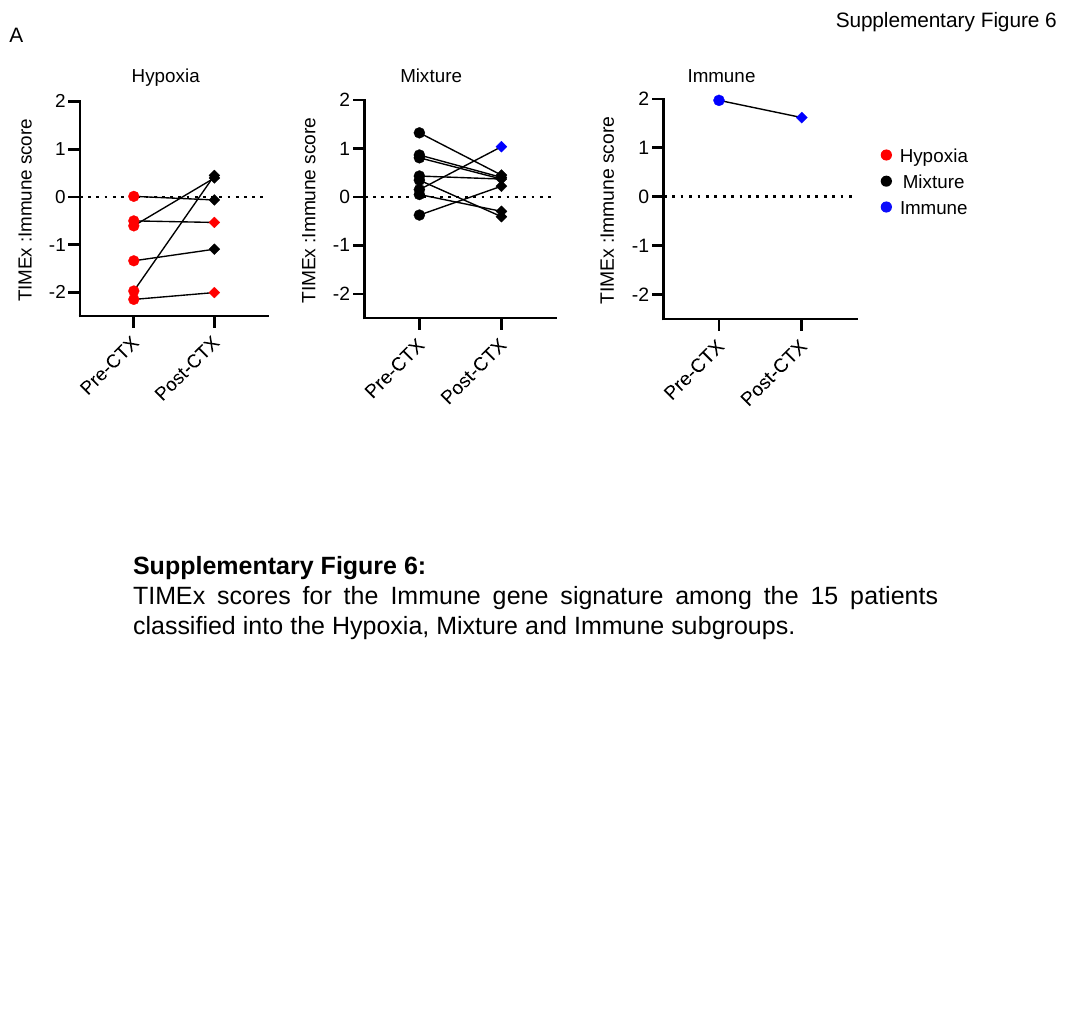

Supplementary Figure 6
A
Hypoxia
Mixture
Immune
Hypoxia
Mixture
Immune
Supplementary Figure 6:
TIMEx scores for the Immune gene signature among the 15 patients classified into the Hypoxia, Mixture and Immune subgroups.
